# Homeostatic Eosinophils: Characteristics and Functions

**DOI:** 10.3389/fmed.2017.00101

**Published:** 2017-07-11

**Authors:** Thomas Marichal, Claire Mesnil, Fabrice Bureau

**Affiliations:** ^1^Laboratory of Cellular and Molecular Immunology, GIGA-Research, University of Liège, Liège, Belgium; ^2^Faculty of Veterinary Medicine, University of Liège, Liège, Belgium; ^3^WELBIO, Walloon Excellence in Life Sciences and Biotechnology, Wallonia, Belgium

**Keywords:** eosinophils, homeostasis, immunomodulation, mucosae, innate immunity

## Abstract

Eosinophils are typically considered to be specialized effector cells that are recruited to the tissues as a result of T helper type 2 (Th2) cell responses associated with helminth infections or allergic diseases such as asthma. Once at the site of injury, eosinophils release their cytotoxic granule proteins as well as preformed cytokines and lipid mediators, contributing to parasite destruction but also to exacerbation of inflammation and tissue damage. Accumulating evidence indicates that, besides their roles in Th2 responses, eosinophils also regulate homeostatic processes at steady state, thereby challenging the exclusive paradigm of the eosinophil as a destructive and inflammatory cell. Indeed, under baseline conditions, eosinophils rapidly leave the bloodstream to enter tissues, mainly the gastrointestinal tract, lungs, adipose tissue, thymus, uterus, and mammary glands, where they regulate a variety of important biological functions, such as immunoregulation, control of glucose homeostasis, protection against obesity, regulation of mammary gland development, and preparation of the uterus for pregnancy. This article provides an overview of the characteristics and functions of these homeostatic eosinophils.

## Introduction

Eosinophils have long been perceived as terminally differentiated cytotoxic and destructive cells that play an effector role mainly in helminthic infections and allergic reactions, such as asthma (1). However, several recent studies have challenged the simplistic view of eosinophils as being exclusively involved in parasite destruction and allergic inflammation. Indeed, at steady state, blood eosinophils rapidly migrate into the gastrointestinal tract, lungs, adipose tissue, thymus, uterus, and mammary glands, where they are now known to exert a variety of essential homeostatic functions (2, 3). In this Mini Review, we summarize the advances in our understanding of the biology (distribution, phenotypic and morphological features, and ontogeny) and functions of these homeostatic eosinophils (hEos).

## Distribution of hEos

In both humans and mice, most hEos are found in the non-esophageal portions of the gastrointestinal tract, where they principally reside in the *lamina propria* of the small intestine (4–7). Depending on the bibliographic source, the numbers of hEos in the gastrointestinal tract of mice are estimated to be 1.5- to 10-fold higher than in the blood (i.e., ranging from 3 × 10^5^ to 2 × 10^6^ cells) (8, 9). Pulmonary hEos are located in the lung parenchyma of both humans and mice (10). In C57BL/6 mice, the numbers of lung hEos exceed 4 × 10^5^, which corresponds to two times the numbers of eosinophils present in the entire circulation (10). In the adipose tissue of mice, hEos account for 4–5% of the stromal/vascular fraction cells (11). In the other organs, hEos reside only transiently (8, 12–16). In mice, the numbers of thymic hEos increase drastically after birth to reach a peak at 2 weeks of age (15). Their numbers then diminish significantly but rise again at 16 weeks when thymic involution starts (15). During the first recruitment phase, hEos concentrate in the cortico-medullary region of the thymus, whereas they are more prominent in the medulla at latter time points (15). It is noteworthy that, in humans, hEos seem to be already present in the thymus of fetuses (14). In rodents, infiltration of the uterus by hEos coincides with the estrus cycle (12, 13). Numerous hEos are indeed observed in the uterus just prior to estrus, during estrus and 1 day postestrus, whereas only few hEos are present during diestrus (12, 13). The vast majority of these cells are located in the endometrium adjacent to the muscular layer (16). In mice, hEos also home to the mammary gland during postnatal development (17). Mammary hEos are principally found around the growing terminal end buds from 3 weeks until 8 weeks of age (17).

**Figure 1:**
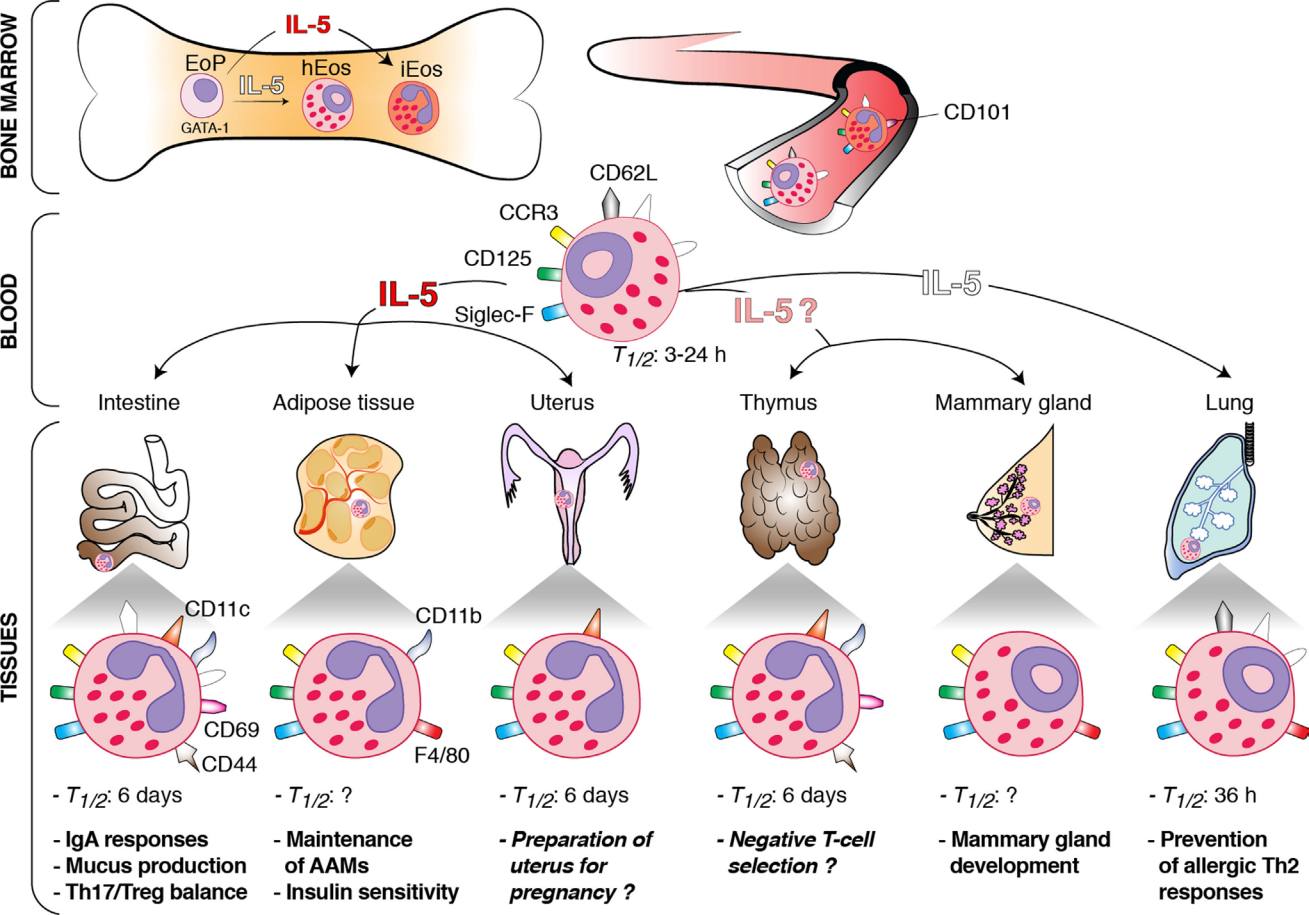
Schematic overview of the origin, interleukin (IL)-5 dependence, phenotype, and functions of homeostatic eosinophils (hEos) in mice. hEos are produced in the bone marrow from the EoP precursor independently of IL-5. Conversely, inflammatory eosinophils (iEos) require IL-5 for their production. hEos are uniformly characterized by expression of Siglec-F, F4/80, CD125, and CCR3. hEos transit through the blood circulation to home into tissues at baseline. Blood hEos have a ring-shaped nucleus and express CD62L, while iEos have a segmented nucleus and do not express CD62L but express CD101. hEos homing to the tissues is either dependent (dark red) or independent (white) on IL-5. The IL-5-(in)dependence of thymic and mammary gland hEos remains unknown. Tissue hEos display distinct phenotype, half-life (*T_1/2_*), and homeostatic functions. The surface phenotype depicted shows whether hEos express (colored symbols) or do not express (white symbols) the indicated surface markers. When marker expression is undefined, the symbol is not present. The function described in *italic* has been suggested, but a clear demonstration is still lacking. h, hours.

*In vivo* studies in humans and mice have shown that eosinophils spend only a short time (i.e., half-life between 3 and 24 h) in the circulation (8, 18, 19). By contrast, hEos remain for a longer time in the tissues. Indeed, their half-life is about 36 h in the lung and up to 6 days in the intestines, thymus, and uterus (8) (Figure 1). The longevity of tissue hEos seems to correlate with CD11c expression. Indeed, while intestine, uterus, and thymus hEos express CD11c, lung, and blood hEos do not express this marker (8, 10) (Figure 1).

Time-course studies in mice have revealed that hEos are not present in the lung at birth but gradually increase in numbers to reach a maximal density by day 7 (10). This observation suggests a link between the colonization of the lung by hEos and the development of the microbiota. Paradoxically, however, hEos recruitment to the gastrointestinal tract seems to be independent of the bacterial flora. Indeed, prenatal mice have detectable hEos in their intestines, and germ-free mice display hEos levels similar to those of control colonized mice (5).

The basal recruitment of hEos to tissues is mainly driven by eotaxin-1 (CCL11), a chemokine produced by local cells such as epithelial cells, endothelial cells, fibroblasts, and monocytes (20–23). Correspondingly, hEos numbers are drastically reduced in the gastrointestinal tract, thymus, and uterus of eotaxin-1-deficient mice (5, 16, 24). Loss of CCR3, the major eotaxin-1 receptor (25, 26), results in defective tissue homing of hEos to the intestines but has no effect on the numbers of lung and thymus hEos (27), which likely relates to the fact that eotaxin-1 may act through alternative receptors such as CCR5 (28). Interleukin (IL)-5 and IL-13, two T helper type 2 (Th2) cytokines, may also promote, although to a lesser extent than eotaxin-1, trafficking of hEos under normal conditions (3, 5). IL-13 enhances eotaxin-1 production (29), while IL-5 supports eosinophil generation from bone marrow progenitors, enhances their sensitivity to eotaxin-1, and sustains their survival (30–32). It has been recently shown that the major source of basal IL-5 and IL-13 in the gastrointestinal tract and the adipose tissue are type 2 innate lymphoid cells (ILC2s) (29, 33). Moreover, after food intake, the vasoactive intestinal peptide stimulates intestinal ILC2 to enhance their secretion of IL-5 and IL-13, linking eosinophil levels with metabolic cycling (29).

## Morphological and Phenotypic Features of hEos

hEos have been mainly characterized in mice. They display most of the typical features of eosinophils, including red staining granules containing toxic cationic proteins (e.g., major basic proteins) and combined expression of CCR3, Siglec-F, and CD125 (i.e., the subunit α of the IL-5 receptor) (8, 9, 34) (Figure 1). They may also express CD11b (intestines, thymus, and adipose tissue), F4/80 (mammary glands, lung, and adipose tissue), CD69 (intestines and thymus), and CD44 (intestines and thymus) (6, 8, 10, 11, 15, 17, 35). In addition, most tissue hEos have a segmented nucleus and express CD11c (8, 13, 15, 16, 24, 35). Lung hEos represent an exception and rather resemble resting blood eosinophils. Indeed, both blood and lung eosinophils have a ring-shaped nucleus (as is the case for mammary hEos as well), express CD62L, display only intermediate levels of Siglec-F, and are negative for CD11c (8, 10, 17, 36, 37) (Figure 1). In mouse eosinophils, such characteristics, especially the presence of a ring-shaped nucleus, are considered a sign of cell immaturity (38, 39), suggesting that pulmonary hEos retain an immature phenotype when spreading into the lungs. However, they undergo piecemeal degranulation and are capable of phagocytosis, demonstrating their functionality (10). Interestingly, the number, localization, and morphological, phenotypic, and transcriptomic features of lung hEos remain unchanged, and differ from those of inflammatory eosinophils (iEos), during allergic airway inflammation (10). iEos, which are abundantly recruited to the lung during airway allergy, are indeed defined as SiglecF^hi^CD62L^−^CD101^hi^ cells with a segmented nucleus (CD101 being an iEos marker that is not expressed by lung hEos) (10). These observations suggest that hEos and iEos represent distinct eosinophil subsets. In line with this hypothesis, hEos- and iEos-like eosinophils are present in the blood of asthmatic mice (10), indicating that the differentiation of both subsets occurs even before their recruitment to the tissues. Furthermore, the parenchymal hEos found in non-asthmatic human lungs (Siglec-8^+^CD62L^+^IL-3R^lo^ cells) are phenotypically distinct from the iEos isolated from the sputa of eosinophilic asthmatic patients (Siglec-8^+^CD62L^lo^IL-3R^hi^ cells), confirming the mouse findings (10).

## Origin of hEos

Eosinophil development depends on a complex interplay of several transcription factors, including GATA-binding protein-1 (GATA-1), CCAAT/enhancer-binding protein-α and -ε (C/EBP-α and -ε), E26 family transcription factor PU.1 (PU.1), and X-box-binding protein-1 (1, 3, 40–42). Among these transcription factors, GATA-1 is the most selective, as attested by the fact that ΔdblGATA mice, in which the double palindromic GATA-1-binding site in the *Gata1* promoter has been genetically deleted, specifically lack eosinophils, including blood and tissue hEos (7, 10, 11, 36, 43). IL-5, which is the most specific cytokine for the eosinophil lineage, is dispensable for the steady-state production of eosinophils. Indeed, the basal numbers of blood eosinophils are only moderately reduced in IL-5-deficient mice, which are, however, unable to develop eosinophilia in the context of a Th2 response (44). Interestingly, recruitment of hEos to the tissues is independent (lungs), partly dependent (gastrointestinal tract and uterus), or entirely dependent (adipose tissue) on local IL-5 production (5, 10, 33, 44, 45) (Figure 1). Given that IL-5 enhances eosinophil survival following migration into the tissues, and that hEos that partly depend on IL-5 (gastrointestinal tract and uterus) have a higher half-life (see Distribution of hEos) than the IL-5-independent ones (lungs), one may speculate that the longevity of tissue hEos is directly linked to their dependence on IL-5. All these observations, if applicable to humans, could also explain why residual eosinophils are found in the blood and lungs of patients treated with anti-α-IL-5 antibodies (46–48).

## Functions of hEos

Depending on the type of tissue they infiltrate, hEos are fulfilling completely different tasks, suggesting the local environment is able to drive hEos functions according to its specific needs. Here, we will review the tissue-specific homeostatic functions of hEos, also summarized in Figure 1.

### Gastrointestinal Tract

Small intestinal hEos are now considered as actively contributing to intestinal homeostasis, allowing the host to cope with the constant and intense exposition to potentially pathogenic microorganisms and foreign and food antigens. In two independent studies, hEos have been shown to be required for the development and maintenance of immunoglobulin (Ig)A-producing plasma cells (7, 35), concordant with the function of bone marrow eosinophils in supporting plasma cell survival (49). They also promote class switching toward secretory IgA, components involved in the neutralization and regulation of intestinal microorganisms (7, 35). In addition, eosinophil deficiency has been associated with altered gut microbiota composition (7, 35), altered development of Peyer’s patches, and decreased mucus production in the small intestine (35), as well as increased numbers of Th17 cells (50) and decreased numbers of regulatory T cells and dendritic cells in gut-associated tissues (7). *In vitro*, Chen and colleagues have shown that small intestinal hEos are able to induce differentiation of naive T cells into Foxp3^+^ regulatory T cells through IL-1β- and retinoic acid-dependent mechanisms (51). More recently, small intestinal hEos have also been shown to suppress the *in vitro* differentiation of Th17 cells and intestinal T cell-derived IL-17 production by secreting large amounts of the IL-1 receptor antagonist IL-1Rα (50). Altogether, these findings are concordant with the idea that small intestinal hEos contribute to intestinal homeostasis by regulating adaptive humoral IgA responses and cellular T cell responses.

### Adipose Tissue

Eosinophils have been emerging as central regulators of adipose tissue metabolism and metabolic health. In adipose tissues, hEos are present together with alternatively activated macrophages (AAMs), and such hEos produce IL-4, thereby favoring the polarization of adipose macrophages toward the alternatively activated phenotype (11). AAMs play a crucial role in glucose homeostasis and development of beige fat, which improves glucose tolerance, insulin reactivity, and, hence, protects against obesity (11, 52, 53). In the absence of adipose hEos, AAMs are greatly reduced and biogenesis of beige fat is impaired (11, 54). Moreover, eosinophil-deficient mice on high-fat diet develop obesity, insulin resistance, and impaired glucose tolerance (11). Conversely, wild-type mice on a high-fat diet but infected with the gastrointestinal nematode *Nippostrongylus brasiliensis*, which triggered a greater eosinophil recruitment in the adipose tissues, exhibit a long-lasting improved sensitivity to insulin and glucose tolerance (11).

This important hEos/macrophage axis is regulated by ILC2s, which sustain adipose hEos and AAMs (33), and is promoted by microbiota depletion (55) and caloric restriction (56).

### Uterus

It is known for decades that hEos infiltrate the non-pregnant uterus of rodents and humans in a cyclic manner, with a peak during estrus (13, 57, 58), but few studies have assessed their potential contribution to the physiology of uterus and to reproductive functions. Gouon-Evans and Pollard examined eotaxin-deficient animals, in which recruitment of hEos to the uterus was impaired, and found a delay in the establishment of the first estrus cycle along with the first age of parturition in those animals compared to wild-type controls (16). While these observations point toward a potential role for hEos in preparing the uterus for pregnancy (16), they must be balanced by the fact that the timing of establishment of subsequent estrus cycles in mature mice is not affected by the absence of eosinophils (16). Most importantly, no fertility issues have been reported in constitutively eosinophil-deficient mice (36, 59), demonstrating that hEos are not essential for normal reproduction.

### Thymus

The presence of thymic hEos in the close vicinity of immature double-negative thymocytes and their abundance in neonates suggest that they may contribute to the process of central tolerance and negative T-cell selection (15). Supporting this, thymic hEos numbers rapidly increase and hEos cluster with apoptotic bodies in an acute model of MHC-I-dependent negative selection (15). Another report proposes that hEos may contribute to the clearance of apoptotic cells, as eosinophil-deficient mice subjected to irradiation-induced thymocyte death are impaired in their ability to phagocyte apoptotic cells (43). However, the definitive proof of a homeostatic role for thymic eosinophils in the process of negative T-cell selection is currently lacking.

### Mammary Gland

A role for eosinophils in regulating postnatal mammary gland development has been proposed in mice (17). Indeed, ablation of hEos recruitment to the mammary glands in eotaxin-deficient animals resulted in a reduced number of branches of the mammary ductal tree and of terminal end buds (i.e., the precursors of alveolar buds) (17). A similar phenotype was observed in the mammary tissue of IL-5-deficient mice as compared to the one from wild-type mice, although the specific contribution of IL-5 itself vs. IL-5-dependent eosinophils has not been assessed in this model (60). Nevertheless, such IL-5-mediated developmental events appear to have functional consequences, as IL-5-deficient nursing dams gave rise to decreased litter size and weanling survival, a phenomenon rescued when IL-5-deficient pups were nursed by IL-5-sufficient dams (60).

### Lungs

Microarray analyses revealed that lung hEos, unlike lung iEos, express several genes, such as *Anxa1, Nedd4, Runx3, Serpinb1a*, and *Ldlr*, that are implicated in the maintenance of lung immune homeostasis, and especially in the negative regulation of Th2 cell responses (10). In line with this observation, eosinophil-deficient ΔdblGATA mice exhibit increased sensitivity to house dust mites (10), confirming that lung hEos are endowed with the capacity to prevent Th2-driven airway allergy. This immunosuppressive function of lung hEos is linked to their unique ability to inhibit the maturation, and therefore the pro-Th2 function, of allergen-loaded dendritic cells (10).

## Conclusion

Although hEos are far from being fully characterized, it is fascinating to see how fast our understanding of the complexity of their phenotype and functions is growing. The fact that these cells exert crucial homeostatic roles at multiple levels merits further investigations and is of medical importance. Indeed, anti-eotaxin-1 and eosinophil-depleting agents, such as humanized anti-IL-5 receptor antibodies and anti-Siglec-8 molecules, are currently being developed to treat eosinophilic disorders such as allergic asthma (9, 61–66), and one has to keep in mind the possibility that such drugs may disrupt tissue homeostasis by preventing organ-specific homing of hEos or by affecting their survival or functions.

## Author Contributions

TM, CM, and FB conducted a review of the literature. TM and FB wrote the manuscript.

## Conflict of Interest Statement

The authors declare that the research was conducted in the absence of any commercial or financial relationships that could be construed as a potential conflict of interest.
